# Standardised Research Methods and Documentation in Cultural Adaptation: The Need, the Potential and Future Steps

**DOI:** 10.32872/cpe.5513

**Published:** 2021-11-23

**Authors:** Eva Heim, Christine Knaevelsrud

**Affiliations:** 1 Institute of Psychology, University of Lausanne, Lausanne, Switzerland; 2 Department of Psychology, University of Zürich, Zürich, Switzerland; 3 Department of Education and Psychology, Freie Universität Berlin, Berlin, Germany; Philipps-University of Marburg, Marburg, Germany

**Keywords:** cultural adaptation, refugees, randomised controlled trials, documentation, monitoring, formative research

## Abstract

**Background:**

Refugees and asylum seekers in Europe are affected by high prevalence of common mental disorders. Under the call ‘mental health of refugee populations’, the German Federal Ministry of Education and Research (FMER) funded a series of research projects to test evidence-based psychological interventions among refugee populations in Germany. In addition, the “Task force for cultural adaptation of mental health interventions for refugees” was established to develop a structured procedure for harmonising and documenting cultural adaptations across the FMER-funded research projects.

**Method:**

A template for documenting cultural adaptations in a standardised manner was developed and completed by researchers in their respective projects. Documentation contained original data from formative research, as well as references and other sources that had been used during the adaptation process. All submitted templates and additional materials were analysed using qualitative content analysis.

**Results:**

Research projects under the FMER call include minors, adults, and families from different origins with common mental disorders. Two studies used and adapted existing manuals for the treatment of PTSD. Four studies adapted existing transdiagnostic manuals, three of which had already been developed with a culture-sensitive focus. Four other studies developed new intervention manuals using evidence-based treatment components. The levels of cultural adaptation varied across studies, ranging from surface adaptations of existing manuals to the development of new, culture-sensitive interventions for refugees.

**Conclusions:**

Cultural adaptation is often an iterative process of piloting, feedback, and further adaptation. Having a documentation system in place from start helps structuring this process and increases transparency.

## Highlights

- A series of evidence-based psychological interventions are tested among refugees in Germany.
- A structured procedure for harmonising and documenting cultural adaptations was developed.
- Cultural adaptation is often an iterative process of piloting, feedback, and further adaptation.
- Documenting the decision-making process, based on evidence from formative research, is key.

In view of the increasing numbers of refugee populations worldwide (UNHCR, 2020) and the high prevalence of common mental disorders among them (Turrini et al., 2017), there is an urgent need for evidence-based mental health interventions to target these populations. According to the World Health Organization (WHO, 2017), common mental disorders include depression, anxiety, and posttraumatic stress disorder (PTSD).

Substantial empirical evidence reveals cultural variation in how symptoms of common mental disorders are expressed (Haroz et al., 2017; Kohrt et al., 2014). In addition, culture-specific assumptions about mental health and mental disorders (e.g., Grupp et al., 2018; Kohrt & Hruschka, 2010) and beliefs about treatment and recovery (e.g., Reich et al., 2015; Shala et al., 2020) have been documented. Based on this evidence, the term *cultural concepts of distress* (CCD) was introduced in the *Diagnostic and Statistical Manual of Mental Disorders*, fifth edition (DSM-5, American Psychiatric Association, 2013). CCD include i) idioms of distress, ii) cultural explanations, and iii) cultural syndromes (Lewis-Fernández & Kirmayer, 2019). Evidence shows that CCD differ from diagnostic categories in the DSM (Kohrt et al., 2014).

There is an ongoing debate on the extent to which psychological interventions developed in Western, Educated, Industrialised, Rich, and Democratic (WEIRD) societies (Henrich et al., 2010) require cultural adaptation to be effective for the treatment of common mental disorders among ethnic and cultural minorities. Ethnic minorities are generally underrepresented in clinical trials in high-income countries (Hussain-Gambles et al., 2004; La Roche & Christopher, 2008; Wendler et al., 2005), which means that the term ‘evidence-based interventions’ has to be used with caution in this context. For this reason, the WHO and other groups of researchers increasingly invest in cultural adaptation of psychological interventions prior to testing them in randomised controlled trials (RCTs) (e.g., Abi Ramia et al., 2018; Heim et al., 2019; Tol et al., 2018).

Resnicow et al. (1999) differentiate between surface and deep structure adaptations. Surface structure adaptations refer to matching materials (e.g., illustrations, language), as well as channels and settings for treatment delivery to observable characteristics of the target population. By contrast, deep structure adaptations convey salience of an intervention by considering how members of a particular cultural group perceive the cause, course, and treatment of a particular illness.

Several meta-analyses showed that culturally adapted psychological interventions are effective when compared to a variety of control conditions (e.g., Chowdhary et al., 2014; Griner & Smith, 2006). One meta-analysis (Hall et al., 2016) found that culturally adapted versions were more effective than the unadapted versions of the same intervention in direct comparison (*g* = 0.52). And one meta-analysis showed that the adaptation of the ‘illness myth’ (i.e., the explanatory model and the treatment rationale) was the most important factor contributing to higher efficacy of adapted interventions (Benish et al., 2011).

Although the meta-analytic evidence seems promising, it is important to mention that it is based on rather low quality of evidence, caused by the following three specific flaws in prior studies: First, there is a lack of theoretical underpinnings in cultural adaptation literature. Most of the literature is based on heuristic frameworks that were developed based on expert opinions and previous studies. A theory-based approach that takes into account literature from the field of cultural clinical psychology and transcultural psychiatry could potentially contribute to a better understanding of *what* to adapt and *why.* Second, previous frameworks for cultural adaptation have focused on clinical practice (e.g., Bernal et al., 2009; Castro et al., 2010; Chu & Leino, 2017; Hwang, 2006), but there are no frameworks or guidelines for implementing and documenting cultural adaptations in psychological trials. This is a particularly relevant gap in the literature, as it hinders the replicability and transparency of trials, both of which are increasingly demanded in the scientific realm. In most published studies, adaptation methods are not well documented (Harper Shehadeh et al., 2016), which leads to a ‘black box’ with regard to *how* the cultural adaptation was implemented. This lack of documentation also adds to blurring sources of bias when assessing and analysing factors of intervention efficacy. Third, and as a consequence of the first two flaws, there is a lack of empirical evidence on the kinds of adaptations that lead to higher acceptability or efficacy of treatments (Heim & Kohrt, 2019). In order to foster empirical research and replicability, transparent criteria on how to implement and document the process of cultural adaption are needed (Heim et al., 2021, this issue).

To address the first problem — the lack of theory — Heim and Kohrt (2019) developed a new conceptual framework for the cultural adaptation of psychological interventions as a basis for empirical research. The authors suggest using CCD as the pivotal element for cultural adaptation and to adapt treatment elements to CCD. Treatment elements are defined in accordance with a taxonomy developed by Singla et al. (2017). This taxonomy differentiates among specific treatment elements (i.e., interventions based on theoretical assumptions such as behavioural or cognitive treatment elements), unspecific treatment elements (i.e., common factors such as the therapeutic relationship or providing a meaningful treatment rationale), and therapeutic techniques (i.e., exercises and other interventions that are done to transmit the therapeutic components, such as role plays or homework). In accordance with Resnicow et al. (1999), adaptations of specific and unspecific treatment elements are deep structure adaptations. With regard to surface adaptations, Heim and Kohrt (2019) suggest considering different modes of treatment delivery (e.g., Internet-based, face-to-face, and group interventions). In addition, surface cultural adaptations include, for example, modifications to texts, illustrations, or case examples.

The present paper addresses the second problem, the lack of documentation. It outlines the work and outcomes achieved by the task force for cultural adaptation of mental health interventions for refugees. This task force was established by a group of researchers from Germany and Switzerland. In 2016, the German Federal Ministry of Education and Research (FMER) launched a call for research proposals covering the ‘mental health of refugee populations’. Seven research projects were funded. One exclusively focuses on diagnostics, and the other six projects will test evidence-based psychological interventions. Each of those six projects consists of three or more sub-projects, in which different interventions with different target groups are tested, implementation methods are compared, and other aspects such as cost-effectiveness are addressed. A total of 11 RCTs (RCTs) will be conducted within these six larger projects. These research projects are currently ongoing. A total of 11 RCTs will be conducted within these six larger projects.

The task force for cultural adaptation of mental health interventions for refugees was launched as a cross-cutting project to harmonise and document cultural adaptation across the 11 sub-projects. The parallel implementation of 11 RCTs that include diverse target populations and a variety of interventions offered a unique opportunity to develop and test such a standardised procedure and to consolidate the experiences in a shared learning process.

Regarding the third problem — the lack of empirical evidence — more consistent documentation of cultural adaptation procedures will contribute to enhancing transparency and replicability in clinical trials. Consistent documentation will also foster meta-analytic evidence, as it will be possible to compare studies with regard to the level (and quality) of cultural adaptation applied in such trials.

## Aim

The task force for cultural adaptation of mental health interventions for refugees aimed to develop a structured procedure for harmonising and documenting cultural adaptations of psychological interventions in clinical trials. The present paper describes the procedures and outcomes of this joint initiative.

## Method

### Procedures

The task force started its work in July 2019. It consisted of the coordinator (first author, EH) and representatives of the 11 RCTs. Representatives were principal investigators and post-doctoral researchers in charge of the cultural adaptation process in each study.

In a first step, the coordinator revised the project descriptions and gathered additional information in telephone interviews with representatives of each project. After these initial contacts, a first workshop was held in September 2019 in which the members of the task force agreed on a common procedure to guide and monitor the cultural adaptation process across the 11 projects. Thereafter, a series of webinars and conference calls was held between October and December 2019 to discuss upcoming topics in the cultural adaptation process. In a second workshop, which was held in February 2020, all members of the task force presented their results of the cultural adaptation process. Experiences were shared and consolidated in small group discussions about specific topics.

### Documentation

A template for documenting cultural adaptations in a standardised manner was developed based on the theoretical framework by Heim and Kohrt (2019). It consisted of the following sections: i) target group; ii) formative research methods; iii) CCD (i.e., idioms of distress, explanatory models); iv) target intervention; v) deep structure adaptations (i.e., specific and unspecific elements, in-session techniques); and vi) surface adaptations (i.e., mode of delivery, materials). Researchers in their respective projects used the template to document the cultural adaptation process. This documentation contained original data (e.g., gathered through key informant interviews of focus group discussions), as well as references and other sources that had been used during the adaptation process (e.g., published papers on CCD in the target population, pilot studies, or formative work). A revised version of the template can be found in Heim et al. (2021), this issue.

### Data Analysis

All submitted templates and additional materials were entered into an NVivo database and analysed using qualitative content analysis. Codes corresponded to the sections of the template (i.e., target group, CCD, elements of the target intervention, etc.). A few sub-codes were developed inductively from the data, where researchers had provided information that did not correspond to one of the sections of the template (e.g., cultural concepts of attachment). Data were analysed by the first author (EH). Since researchers themselves had allocated information on their projects to the corresponding sections and sub-sections of the template, no second coder was involved in the data analysis. Disagreements were clarified between the first author and researchers who had completed the template.

## Results

### Overview of Studies

An overview of the 11 projects is provided in Table 1 in the Appendix, Supplementary Materials). Eight of the 11 sub-projects returned the completed templates and additional material. The other three had already completed the cultural adaptation, with limited possibilities to document this process. In four studies, the process of cultural adaptation was documented retrospectively by analysing qualitative data collected during the adaptation process that had not been analysed nor published. And four studies adapted their interventions during the course of the present project and documented this process continuously.

### Target Populations and Disorders

The first section of the template contained the target population and the ‘Western’ diagnostic categories addressed in the respective trials. Three studies focused on minors, five on adults, and two on families (i.e., parents and their children). The majority (seven studies) included refugees from different countries of origin, three studies included Afghan and Syrian refugees, and one study included Arabic-speaking refugees. Studies including refugees from different countries of origin developed a ‘culture-sensitive’ rather than a ‘culture-specific’ treatment approach (e.g., Lotzin et al., 2021, this issue). Across the 11 projects, the targeted disorders were post-traumatic symptoms (five studies), common mental disorders (three studies as primary outcome, and one study as secondary outcome), and substance use disorder (one study). In addition, one study aimed at increasing knowledge about common mental disorders, psychological resources, and services of care (Mewes et al., 2021, this issue).

### Formative Research Methods

Researchers in the respective projects gathered relevant information for cultural adaptation from published literature and through qualitative research. Formative research revealed information related to CCD, as well as information regarding the target interventions themselves (e.g., acceptance, suggestions for adaptations). A literature review was conducted in all but one project. In three studies, information on CCD (i.e., idioms of distress and explanatory models) was gathered through focus group discussions or individual interviews ahead of starting the process of cultural adaptation.

### Cultural Concepts of Distress

Results of literature reviews and qualitative research revealed mind–body concepts, idioms of distress, and explanatory models, which are described more in detail in the respective papers in this special issue. In addition to CCD, other related concepts were taken into account: Two studies considered assumptions about help-seeking, and one study reported on cultural concepts of attachment. In addition, four studies reported that fatalistic beliefs were relevant in their target populations. And one study also included cultural resources alongside CCD.

### Target Interventions

The studies varied with regard to their therapeutic approaches. Two studies used and adapted existing manuals for the treatment of PTSD. Four studies adapted existing transdiagnostic manuals, three of which had already been developed with a culture-sensitive focus. Four other studies developed new intervention manuals using evidence-based treatment components.

### Cultural Adaptations: Surface and Deep Structure

The levels of cultural adaptation varied across studies, ranging from surface adaptations of existing manuals to the development of new, culture-sensitive interventions for refugees. Other studies conducted deep structure adaptations of existing interventions, such as by adding, changing, or modifying specific treatment components. In addition, several studies considered the mode of delivery of the intervention, such as online vs. face-to-face, or group vs. individual. Most studies described surface adaptations (Resnicow et al., 1999), such as the use of a culture-specific or culture-sensitive language, the inclusion of specific idioms of distress, the use of illustrations and non-verbal material for non-German speaking participants, or the consideration of gender-related aspects and religious concerns (e.g., not offering food in a closing ritual during Ramadan).

Psychoeducation materials were culturally adapted in most studies, and psychoeducation was extensively discussed during the second workshop of the task force. Some of the considerations around psychoeducation involved metaphors and analogies to describe the therapeutic process, such as the wound metaphor (trauma as a wound), the process of healing (e.g., of a broken leg), psychotherapy as training (e.g., muscle training), or the metaphor of the messy cupboard that must be cleaned up. Another relevant issue in psychoeducation concerned mental health-related stigma, which was addressed in some studies through normalising, the use of non-stigmatising terms, or using an encouraging rather than a problematising language.

Regarding specific therapeutic elements, all studies conducted a careful analysis of interventions to address the most common symptoms of psychological distress in their respective target populations. As an example, three studies discussed the inclusion or exclusion of problem solving in their respective interventions (Böttche et al., 2021, this issue; Kananian et al., 2021, this issue; Unterhitzenberger et al., 2021, this issue). This discussion showed that, on the one hand, problem solving seemed to be a helpful intervention to address the refugees’ most pressing concerns, such as asylum status or family reunification. On the other hand, problem solving seemed to be overly cognitive for some participants, and it bears the risk that such practical problems become more important than the psychotherapeutic work in the sessions. The discussion across the three projects contributed to finding ways to use problem solving while keeping these downsides to a minimum.

### Documentation Process

The level of detail of information provided in the adaptation template varied across studies. Researchers in two studies used the template to guide and document their process of cultural adaptation, whereas two other studies (and four sub-studies) used it to structure the documentation process retrospectively. This was mainly because in these projects, an iterative process of intervention development, cultural adaptation, and piloting had been implemented before acquiring the funding for the RCTs, and hence before the task force had started its work.

During the project, it became clear that the template required more detailed instructions on the information required in the sub-sections. In addition, researchers expressed difficulties in making decisions about cultural adaptations based on the evidence they had gathered on their target population. Two studies specifically focused on this decision-making process from formative research to adaptation (i.e., Böttche et al., 2021, this issue; Lotzin et al., 2021, this issue). Only one study documented the decision-making process itself, that is, the opinions expressed by the different researchers on the team.

## Discussion

Despite the growing body of literature on cultural adaptation of psychological interventions, there is still a lack of evidence on adaptations that will contribute to increase the feasibility, acceptance, and efficacy of such interventions. We have argued that this is mainly due to a lack of theory-based approaches to cultural adaptation (Heim & Kohrt, 2019), of systematic documentation (Harper Shehadeh et al., 2016), and of rigorous empirical studies.

In this project, a standardised documentation procedure was developed and applied across 11 studies that will evaluate psychological interventions in clinical trials with refugee populations in Germany. The parallel implementation of 11 RCTs with refugee populations provided a unique opportunity to develop and test such a standardised procedure and to better understand the process, challenges, and specific requirements of cultural adaptation in psychological trials. Experiences in this project revealed important lessons learned concerning the content (i.e., *what*) and the process (i.e., *how*) of cultural adaptation.

Regarding content, researchers in this task force described both surface and deep structure adaptations (Resnicow et al., 1999). Surface adaptations are increasingly described in the literature (e.g., Chowdhary et al., 2014). Deep structure adaptations, such as the selection or development of treatment elements in accordance with CCD and other relevant aspects, is less prominent in the literature (Hall et al., 2016). Based on the theoretical framework by Heim and Kohrt (2019), several studies included here used CCD (i.e., idioms of distress, explanatory models, and culturally salient symptoms) as a starting point for cultural adaptation. Aside from CCD, other aspects that are relevant for cultural adaptation were mentioned, such as cultural resources (Mewes et al., 2021, this issue), gender and religious aspects (Kananian et al., 2021, this issue), or cultural concepts other than CCD (e.g., attachment). Researchers considered the use of specific treatment elements in function of participants’ needs and conditions, such as problem solving (Böttche et al., 2021, this issue; Kananian et al., 2021, this issue).

Regarding process, our experiences showed that the documentation system must be in place from the beginning of the adaptation process. In three studies, the adaptation was done in parallel with the work of the task force. In other studies, the adaptation process was documented retrospectively based on unpublished data, and some projects had already completed their adaptation process, with limited possibilities for retrospective documentation. It became clear that retrospective documentation is very difficult, even if unpublished data are available (e.g., transcripts from focus groups), particularly due to the difficulty to replicate the decision-making process. In addition, our experiences showed that the template for documentation should be simple and contain clear instructions to avoid additional workload for the research staff.

Decision making is a major challenge in cultural adaptation. Only one study documented the different views of the team members as a basis for decisions (Lotzin et al., 2021, this issue). Another study mentioned the risk of excluding other groups if adaptations are too specific for one particular group (Böttche et al., 2021, this issue). Our experiences show that the decision of *what* to adapt, and *why*, remains a subjective process to some extent. In view of transparency, it is therefore essential to document the considerations behind this process, the different views of team members, and the strength of evidence that supported decisions.

That said, a further process-related lesson learned is that cultural adaptation takes time. Several studies went through an iterative process of piloting, feedback, and further adaptation. Some of the projects used culture-sensitive interventions and further adapted them to their target population (Böttche et al., 2021, this issue; Kananian et al., 2021, this issue; Lotzin et al., 2021, this issue). Keeping track of this process and documenting the different stages of adaptation is a labour-intensive and time-consuming process. One study concluded that the balance between investment (i.e., time and financial expenditure) and outcome was not yet determined (Böttche et al., 2021, this issue). Indeed, experimental studies are needed to determine the effects of cultural adaptation on the feasibility and effectiveness of interventions (Heim et al., 2020).

The present paper has several limitations. First, due to administrative reasons, not all project included in the task force were in the same adaptation phase when the task force started its activities. Some projects had concluded their cultural adaptation process, while other studies conducted the cultural adaptation as part of the task force activities. However, this allowed for considering challenges occurring at different moments throughout the cultural adaptation process, which enhanced the richness of our lessons learned. Second, all studies included in this task force were conducted in Germany, which limits the generalisability to other contexts. And third, this task force focused on the process of documentation only. Examining the effect of cultural adaptation on trial efficacy was beyond the scope of this task force. The main focus of this task force was on establishing a standardised documentation system, which will hopefully be an important step in guiding and improving the quality of cultural adaptation research in the future.

Based on our experiences, a sub-group of the task force elaborated a set of REporting Criteria for cultural Adaptation in Psychological Trials (RECAPT), which are presented in this special issue (Heim et al., 2021, this issue). As a next step, experimental research is needed to determine the impact of surface and deep structure adaptations on the acceptability and effectiveness of psychological interventions. Such experimental research may include RCTs comparing different levels of cultural adaptation (Heim et al., 2020) or other research designs like factorial experiments. In addition, standardised documentation of cultural adaptation can contribute to meta-analytic evidence, in which the association between levels of cultural adaptation and trial effectiveness is analysed in meta-regression (e.g., Harper Shehadeh et al., 2016).

In view of the increasing need to develop and test psychological interventions for diverse cultural and ethnic groups, cultural adaptation can no longer remain the unwanted stepchild in psychological science. Over the past decades, high-quality standards have been increasingly applied in clinical trials in general, which are defined in the CONSORT statement (Moher et al., 2001). Transparency and replicability are increasingly demanded for clinical trials with psychological interventions, not least as a consequence of the open science movement. It is essential that we request the same level of quality, transparency, and replicability for cultural adaptation in clinical trials with culturally diverse groups and ethnic minorities. By using such high-quality standards, the interventions we develop will hopefully be used, have a positive effect, and help people manage their lives.

## Supplementary Materials

The Supplementary Materials contain the following items (for access see Index of Supplementary Materials below):

- **Appendix**
  Appendix A provides an overview table of the research projects described in this paper, i.e., target populations, target symptoms and disorders, interventions, and information on the cultural adaptation process.
- **RECAPT Template**
  A template for documenting the cultural adaptation process that was developed by the “Task force for cultural adaptation of mental health interventions for refugees”. A documented version for better understanding is provided, along with an empty template in Word format that can be used for future studies.

10.23668/psycharchives.5200 Supplement 1 Supplementary materials to "Standardised research methods and documentation in cultural adaptation: The need, the potential and future steps" [Appendix]

10.23668/psycharchives.5192 Supplement 2 Supplementary materials to "Standardised research methods and documentation in cultural adaptation: The need, the potential and future steps" [RECAPT Template]

HeimE.
KnaevelsrudC.
 (2021a). Supplementary materials to "Standardised research methods and documentation in cultural adaptation: The need, the potential and future steps"
[Appendix]. PsychOpen. 10.23668/psycharchives.5200
PMC967083336405674

HeimE.
MewesR.
Abi RamiaJ.
GlaesmerH.
HallB.
Harper ShehadehM.
ÜnlüB.
KananianS.
KohrtB. A.
Lechner-MeichsnerF.
LotzinA.
MoroM. R.
RadjackR.
Salamanca-SanabriaA.
SinglaD. R.
StarckA.
SturmG.
TolW.
WeiseC.
KnaevelsrudC.
 (2021b). Supplementary materials to "Reporting Cultural Adaptation in Psychological Trials – The RECAPT criteria"
[RECAPT Template]. PsychOpen. 10.23668/psycharchives.5192
PMC967082636405678
