# Supplementary material for: Standardised Research Methods and Documentation in Cultural Adaptation: The Need, the Potential and Future Steps
Source: Clin Psychol Eur. 2021 Nov 23;3(Spec Issue):e5513. doi: 10.32872/cpe.5513 (PMC9670833; doi:10.32872/cpe.5513)
Supplement: Supplement 1 [file cpe-03-5513-s01.pdf]

Appendix A

Table 1. Overview of projects

Project	Sub-project	Target population	Target symptoms / disorder	Target intervention	Development vs. adaptation	Documentation	Source of evidence for cultural adaptation
BETTER CARE	S2	Refugee minors (12-21 years old) from different countries of origin who resettled in Germany.	Post-traumatic stress symptoms, depression	"Mein Weg" (English: My Way)<br>(English: My Way; Pfeiffer & Goldbeck, 2019)	Development	Retrospectively	• Feedback from participants and child welfare staff in pilot study (Pfeiffer & Goldbeck, 2017)<br>• Randomised controlled trial (Pfeiffer et al., 2018)<br>• Implementation study (Pfeiffer et al., 2019)
	S3	Unaccompanied young refugees in Germany, no limitation with regard to country of origin or language.	PTSD (primary outcome), other common mental disorders (secondary outcome)	Trauma-focused cognitive behavioral therapy (TF-CBT; Cohen et al., 2017).	Adaptation	Retrospectively	• Literature review<br>• Interviews at outpatient clinic<br>• Case series<br>• Pilot study on TF-CBT with unaccompanied refugee minors

**Project**	**Sub-project**	**Target population**	**Target symptoms / disorder**	**Target intervention**	**Development vs. adaptation**	**Documentation**	**Source of evidence for cultural adaptation**
							(Unterhitzenberger et al., 2019)<br>• Feedback from therapists and interpreters
I-REACH	SP2	Adult Arabic- and Farsi-speaking refugees in Germany	Common mental disorders	Common elements treatment approach (CETA; Murray et al., 2014)	Adaptation	Continuously	• Literature review<br>• Qualitative interviews<br>• Checklists<br>• Focus group<br>• Expert discussions
IMPROVE-MH	N/A	Arabic-speaking refugee families, i.e., parents of children age 0-6	Common mental disorders	Short CBT-based intervention provided by general practitioners	Development	Continuously	• Literature review<br>• Expert discussions<br>• Feedback from psychology students / psychologists with Arab cultural background<br>• Feedback of therapists and participants during the pilot

**Project**	**Sub-project**	**Target population**	**Target symptoms / disorder**	**Target intervention**	**Development vs. adaptation**	**Documentation**	**Source of evidence for cultural adaptation**
PREPARE	N/A	Adult Afghan and Syrian refugees in Germany	Hazardous substance use or substance use disorders	Skills Training in Affect Regulation - a Culture-sensitive Approach (Koch et al., 2020)	Adaptation	Continuously	• Literature review<br>• Focus groups<br>• Feedback of therapists
ReCAP	S1	Adult asylum seekers who recently arrived in Germany from different countries of origin.	Knowledge about symptoms of common mental disorders, psychological resources, and mental health care	'Teagarden' (German: Gesundheits-Teegarten) (Mewes et al., 2015)	Development	Retrospectively	• Literature review<br>• Feedback from therapists<br>• First authors' scientific and clinical work<br>• Experiences of members of the study team (i.e., refugees and persons from different countries of origin and different cultural backgrounds)
	S2	Adult Afghan and Syrian refugees in Germany	Common mental disorders	Culturally adapted cognitive behavioral	Adaptation	Retrospectively	• Literature review<br>• Key informant interviews

Project	Sub-project	Target population	Target symptoms / disorder	Target intervention	Development vs. adaptation	Documentation	Source of evidence for cultural adaptation
				therapy (CA-CBT; Hinton et al., 2012)			• Focus groups after pilot study
	S3	Adult refugees from different countries of origin	PTSD	Brief Imagery Rescripting (Arntz, 2012)	Adaptation	N/A	• Literature review<br>• Feedback from therapists and experts
START	A1	Unaccompanied minor refugees in Germany (age 13-17), languages: English, German, Dari, Arabic, Somali	PTSD	Stabilization and arousal modulation for intensely stressed children and adolescents and minor refugees (START, Dixius & Möhler, 2018)	Development	Retrospectively	• Literature review<br>• Focus groups
	A2	Children and their parents with a refugee background; educators in childcare facilities	High risk for PTSD	Mentalization-based and attachment-orientated educator program (Bark et al., 2016; Mayer et al., 2019)	Adaptation	Continuously	• Literature review

**Project**	**Sub-project**	**Target population**	**Target symptoms / disorder**	**Target intervention**	**Development vs. adaptation**	**Documentation**	**Source of evidence for cultural adaptation**
	A3	Asylum applicants and asylum granted refugees (18-25 years), languages: German and English	PTSD and Disorder of Extreme Stress not Otherwise Specified (DESNOS)	Age-adapted START (Dixius & Möhler, 2018) and integrated biofeedback	Adaptation	Retrospectively	• Literature review

References

Arntz, A. (2012, 2012/04/01). Imagery rescripting as a therapeutic technique: review of clinical trials, basic studies, and research agenda. *Journal of Experimental Psychopathology, 3*(2), 189-208. https://doi.org/10.5127/jep.024211

Bark, C., Baukhage, I., & Cierpka, M. (2016, 2016/03/01/). A mentalization-based primary prevention program for stress prevention during the transition from family care to day care. *Mental Health & Prevention, 4*(1), 49-55. https://doi.org/https://doi.org/10.1016/j.mhp.2015.12.002

Cohen, J. A., Mannarino, A., & Deblinger, E. (2017). *Treating trauma and traumatic grief in children and adolescents, second edition (2nd ed.)*. Guilford Publications.

Dixius, A., & Möhler, E. (2018, 2018/10/01). Stress-Traumasymptoms-Arousal-Regulation-Treatment (START). *Pädiatrie & Pädologie, 53*(1), 34-38. https://doi.org/10.1007/s00608-018-0585-2

Hinton, D. E., Rivera, E. I., Hofmann, S. G., Barlow, D. H., & Otto, M. W. (2012, Apr). Adapting CBT for traumatized refugees and ethnic minority patients: examples from culturally adapted CBT (CA-CBT). *Transcultural Psychiatry, 49*(2), 340-365. https://doi.org/10.1177/1363461512441595

Koch, T., Ehring, T., & Liedl, A. (2020, 2020/09/01/). Effectiveness of a transdiagnostic group intervention to enhance emotion regulation in young Afghan refugees: A pilot randomized controlled study. *Behaviour Research and Therapy, 132*, 103689. https://doi.org/https://doi.org/10.1016/j.brat.2020.103689

Mayer, A., Taubner, S., Bark, C., & Holl, J. (2019, 2019/12/03). Herausforderungen in der frühpädagogischen Arbeit mit geflüchteten Familien mentalisierungsbasiert begegnen. [Mentalization-based encounter to challenges in early childhood education with refugee families]. *Praxis der Kinderpsychologie und Kinderpsychiatrie, 68*(8), 711-727. https://doi.org/10.13109/prkk.2019.68.8.711

Mewes, R., Reich, H., & Demir, S. (2015). *Beratung nach Flucht und Migration. Ein Handbuch zur psychologischen Erstbetreuung von Geflüchteten*. WeltTrends.

Murray, L. K., Dorsey, S., Haroz, E., Lee, C., Alsiary, M. M., Haydary, A., Weiss, W. M., & Bolton, P. (2014). A Common Elements Treatment Approach for adult mental health problems in low- and middle-income countries. *Cogn Behav Pract, 21*(2), 111-123. https://doi.org/10.1016/j.cbpra.2013.06.005

Pfeiffer, E., & Goldbeck, L. (2017, Oct). Evaluation of a trauma-focused group intervention for unaccompanied young refugees: a pilot study. *Journal of Trauma Stress, 30*(5), 531-536. https://doi.org/10.1002/jts.22218

Pfeiffer, E., & Goldbeck, L. (2019). *Traumafokussierte pädagogische Gruppenintervention für junge Flüchtlinge [Trauma-focused educational group intervention for young refugees]*. Hogrefe.

Pfeiffer, E., Sachser, C., Rohlmann, F., & Goldbeck, L. (2018, Nov). Effectiveness of a trauma-focused group intervention for young refugees: a randomized controlled trial. *Journal of Child Psychology and Psychiatry, 59*(11), 1171-1179. https://doi.org/10.1111/jcpp.12908

Pfeiffer, E., Sachser, C., Tutus, D., Fegert, J. M., & Plener, P. L. (2019, 2019/04/01). Trauma-focused group intervention for unaccompanied young refugees: "Mein Weg"—predictors of treatment outcomes and sustainability of treatment effects. *Child and*

Adolescent Psychiatry and Mental Health, 13(1), 18. https://doi.org/10.1186/s13034-019-0277-0

Unterhitzenberger, J., Wintersohl, S., Lang, M., König, J., & Rosner, R. (2019, 2019/05/17). Providing manualized individual trauma-focused CBT to unaccompanied refugee minors with uncertain residence status: a pilot study. *Child and Adolescent Psychiatry and Mental Health, 13*(1), 22. https://doi.org/10.1186/s13034-019-0282-3
